# Short-stretch inelastic compression bandage in knee swelling following total knee arthroplasty study (STICKS): study protocol for a randomised controlled feasibility study

**DOI:** 10.1186/s13063-015-0618-0

**Published:** 2015-03-11

**Authors:** Timothy M Brock, Andrew P Sprowson, Scott Muller, Mike R Reed

**Affiliations:** NIHR academic clinical fellow and orthopaedic registrar, Wansbeck General Hospital, Woodhorn Lane, Northumberland, NE63 9JJ UK; Consultant Orthopaedic Surgeon, Warwick Medical School, University of Warwick, Coventry, CV4 7AL UK; Consultant orthopaedic surgeon, Wansbeck General Hospital, Woodhorn Lane, Northumberland, NE63 9JJ UK

**Keywords:** Knee replacement, Arthroplasty, Compression bandage, Enhanced recovery, Fast track

## Abstract

**Background:**

Enhanced recovery programmes in total knee arthroplasty are well established. Post-operative knee swelling is common and impairs early post-operative function. The use of a short-stretch, inelastic compression bandage is hypothesised to reduce knee swelling and improve pain and early function. A study was designed to test feasibility with a view to informing a larger, future trial and to provide preliminary data.

**Methods/design:**

This is a randomised controlled feasibility study. Fifty consecutive patients selected for primary total knee arthroplasty will be enrolled in the trial. Patients with a BMI >35, latex allergy or neurological or peripheral vascular disease are excluded. Patients are randomised by distance randomisation to receive a compression bandage for 24 hours after surgery or a standard wool and crepe bandage. The bandages are applied by one of two consultant surgeons who have had training with their application. Knee swelling, range of motion and pain scores will be compared pre-operatively and at day 1, day 2 and at 6 weeks between groups. The Oxford knee score and EQ-5D health status will be compared pre-operatively and at 6 months between groups. Recruitment rates, retention rates, resource allocation, completeness of data collection, and tolerance and complications with the compression bandage are recorded. Descriptive statistics are used to calculate a standard deviation for post-operative knee swelling in the groups and to perform a power calculation incorporating anticipated patient retention rates to inform a future trial. Preliminary data will be analysed using the independent samples *t*-test for equal distributions and the Mann-Whitney U for unequal distributions with the significance denoted at *P* <0.05.

**Discussion:**

Enhanced recovery programmes have revolutionized the management of total knee arthroplasty. There is a paucity of clinical data regarding the efficacy of compression bandages. Large, randomised controlled trials are uncommon in orthopaedic surgery. The results of this study will provide feasibility and preliminary data prior to the construction of a larger, multicentre study.

**Trial registration:**

The study was registered with Current Controlled Trials (ISRCTN86903140) on 30 May 2013.

## Background

Total knee replacement is a common and highly successful operation in the management of osteoarthritis. The evolution of enhanced recovery programmes over the last 15 years has reduced hospital stay and morbidity, without increasing readmission rates [1,2].

Post-operative knee swelling is a common problem following total knee replacement. This is largely due to intra-articular bleeding and inflammation of periarticular tissues [3]. Knee swelling results in decreased functional performance as a result of quadriceps weakness [4] and as a result of arthrogenic reflex inhibition due to pain [5]. The resulting decrease in functional performance can delay rehabilitation and affect length of stay and patient-reported outcomes [6,7]. Excessive knee swelling is also associated with increased rates of dehiscence and infection in surgical wounds [8].

Various techniques have been utilised to reduce intra-articular bleeding, including modification of surgical technique [9], tourniquets [10], medication [11] and rehabilitation protocols [12]. Post-operative methods include the use of a cold compress [13], cryotherapy [14], elastic bandaging [15] and compression bandages [16,17].

However, there is a paucity of clinical data regarding the use of compression bandages with heterogeneous methodology. Andersen *et al*. utilised a compression bandage consisting of a double layer of soft padding and an outer elastic adhesive dressing compared with standard bandage in a randomised controlled trial of 48 patients [16]. The bandage was combined with local anaesthetic infiltration. The intervention group had significantly reduced pain at 8 hours compared to a control group. There was no difference in length of stay. As this was an acute pain study, range of motion, swelling and quality of life were not assessed.

A randomised controlled trial of 60 patients undergoing unicondylar knee arthroplasty found no difference in swelling and post-operative pain in patients who received a modified Robert-Jones type bandage compared to a standard wool and crepe bandage when assessed at 24 h and 48 h post-operatively [18]. Conversely, a non-randomised study of 150 patients undergoing total knee arthroplasty found a compression bandage to be advantageous, with improvements in total range of motion and length of stay when compared to wool and crepe bandages [12].

Munk *et al*. randomised 88 patients to receive either an elastic medical compression stocking or no stocking for 4 weeks after total knee arthroplasty [19]. The main outcome measures were knee swelling, knee flexion and pain scores. The study did not find any clinical effect between the two groups. However, the stocking was applied the day after surgery when it was documented that 70% of the swelling had already occurred.

Therefore, the efficacy of compression bandage therapy after total knee arthroplasty remains unclear due to varying methodology and small sample sizes. The efficacy of compression bandaging in the treatment of venous ulcers and lymphoedema is encouraging [20,21]. With regard to total knee arthroplasty, there are two main mechanisms that cause post-operative swelling. The first is an increase in vascular permeability as a result of the release of histamine and histamine-like substances causing the capillary network to allow more electrolytes and plasma proteins into the tissues as a direct response to trauma [8]. This causes an imbalance in homeostasis and a net movement of fluid into the interstitial space from the vascular space. This response is short lived but deleterious. Secondly, vascular injury from the surgery causes bleeding, which leaks into the tissues until plugged by thrombus [8]. Whilst the mechanisms of compression therapy are poorly understood, it is believed that the application of external compression causes a decrease in hydrostatic pressure by aiding venous return as a result of moving blood from the superficial to deep venous system and by improving the efficacy of the calf muscle pump. This allows movement of fluid from the interstitial space. It also aids lymphatic drainage, and a resultant hyperaemic response improves arterial blood flow [22]. With regard to the type of bandage, inelastic compression bandages have been found to have a low, tolerable resting pressure but a more effective activation of the deep venous system and calf muscle pump with ambulation compared to the elastic counterparts [23].

It is hypothesised that the use of a compression bandage in total knee arthroplasty will therefore reduce lower leg swelling by improving venous return post-operatively. This improvement will improve knee extension strength and reduce pain inhibition, allowing earlier, effective rehabilitation and an improvement in length of stay and patient reported outcome measures.

To test this hypothesis, a large, randomised controlled trial is required. The aim of this current study is to test the feasibility of the methodology and gather data to inform a larger future trial. The secondary objective is to perform a small-scale trial to provide preliminary data.

## Methods/design

The study is a prospectively enrolled, randomised controlled feasibility study conducted at two hospital sites within the Northumbria Healthcare NHS Foundation Trust. The study has been approved by the Newcastle and North Tyneside Research Ethics Committee (13/NE/0137), and all patients will provide written, informed consent. The study has been registered with Current Controlled Trials (ISRCTN86903140) and is sponsored by Northumbria Healthcare NHS Foundation Trust.

### Participant recruitment

A total of fifty consecutive patients selected for total knee arthroplasty will be enrolled in the study. Due to insufficient data in the literature to determine a clinically relevant change in outcome measures, the sample size is based on the ability to calculate a mean and standard deviation in these measures to allow an accurate power calculation for the future trial. The sample size conforms to guidelines on feasibility studies in the literature and is large enough to additionally provide preliminary data [24].

### Study duration

The study started recruiting in November 2013, and data collection is expected to finish in December 2014. This timeframe is based on 2 months initial set-up, 6 months recruitment and 6 months follow-up. Six months recruitment assumes approximately two patients per week or one patient per operating surgeon. Based on joint registry data, this assumes an approximate inclusion rate of 66% based on the two operating surgeons’ caseloads.

### Inclusion /exclusion criteria

The inclusion criteria for the study include (i) primary total knee arthroplasty for osteoarthritis, (ii) age over 18, and (iii) able to provide written, informed consent.

Exclusion criteria include (i) peripheral vascular disease characterised by an ABPI <0.8, (ii) peripheral neuropathy, (iii) BMI >35, (iv) revision knee arthroplasty, (v) unicondylar or patellofemoral joint knee arthroplasty or (vi) high dose anticoagulation. The majority of criteria have been established to reduce confounding variables associated with post-operative swelling in knee arthroplasty or due to contraindications with the compression bandage.

### Consent

The patient is given an information sheet regarding the trial after being listed for knee joint arthroplasty. Any questions are answered by a good clinical practice (GCP)-trained research nurse before the patient is consented into the trial. The patient is given at least 24 hours to make this decision following initial supply of information.

### Patient allocation

The patient is randomised to the intervention on the day of surgery by electronic distance randomisation using the website www.sealedenvelope.com. The randomisation process is done in permuted blocks to allow even balancing of the groups. Patients will be allocated to the standard care group or the compression bandage group as outlined below.

### Standard care group

**Figure 1:**
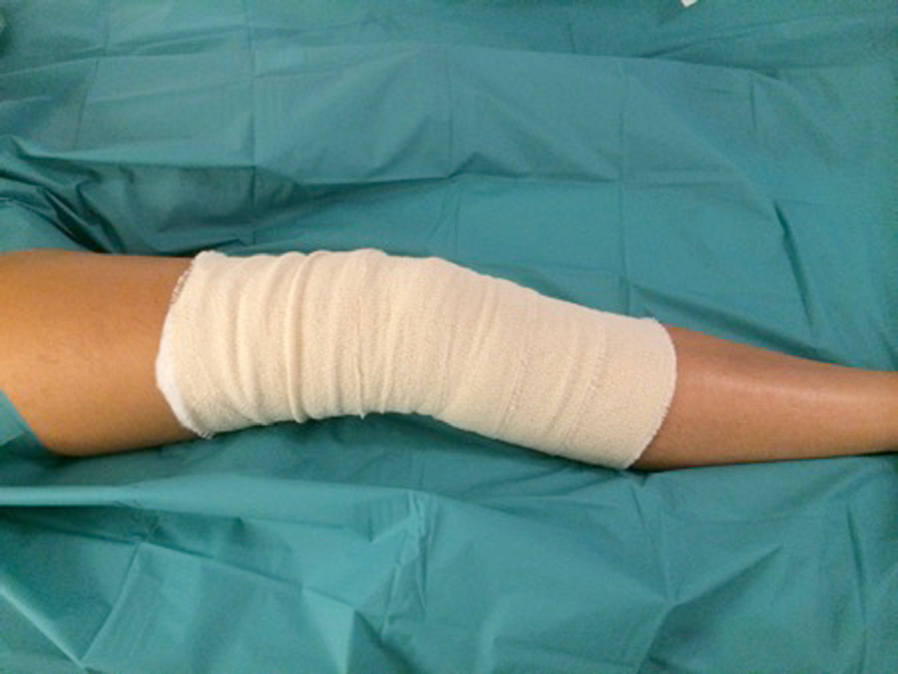
**Standard wool and crepe bandage.**

Patients undergo primary total knee arthroplasty under the care of one of two consultant orthopaedic surgeons (SM, MR). Surgery is with either general anaesthesia or spinal anaesthesia and sedation. Intravenous antibiotics (gentamicin 3 mg/kg and teicoplanin 400 mg iv) and tranexamic acid (30 mg/kg iv up to 2.5 g) are administered and a tourniquet is used. A Nexgen cruciate retaining total knee arthroplasty is routinely used for primary total knee arthroplasty in the trust (Zimmer, Swindon, United Kingdom) with Palacos R + G bone cement (Heraeus Medical, Newbury, United Kingdom). Intra-operative periarticular injections of 80 ml 0.125% bupivacaine are infiltrated, and a further 20 ml 0.125% bupivicaine bolus is given via intra-articular wound catheter after wound closure. The skin is closed using surgical skin clips, which are removed at 10 to 14 days post-operatively. A hydrocolloid dressing (Aquacel surgical, Convatec Ltd, Flintfield, UK) is used for the wound. Standard bandaging consists of a soft inner layer (Soffban, BSN Medical Ltd, Brierfield, UK) applied from 10 cm below to 10 cm above the patella with a 50% overlap of bandage, followed by a similar outer layer of crepe bandage (BSN Medical Ltd, Brierfield UK) prior to deflation of the tourniquet (Figure 1).

The bandage and wound catheter are removed at 24 hour leaving the hydrocolloid wound dressing *in-situ*. This dressing stays on until the clips are removed at 10 to 14 days and prevents any contamination of the wound, for example, during the knee swelling measurements. A cryocuff is used after 24 hours.

Physiotherapy starts on the day of surgery. Focus initially is on first time mobilisation, quadriceps strengthening exercises and passive full extension. Following this, patients are seen at least twice daily by the physiotherapist, and range of motion, gait re-education and lower limb strengthening techniques are employed every day until discharge from hospital. Patients are seen weekly in an outpatient physiotherapy class if required until they are independently walking, have full passive extension and a range of motion in the knee from 0 to 90 degrees.

### Intervention group

**Figure 2:**
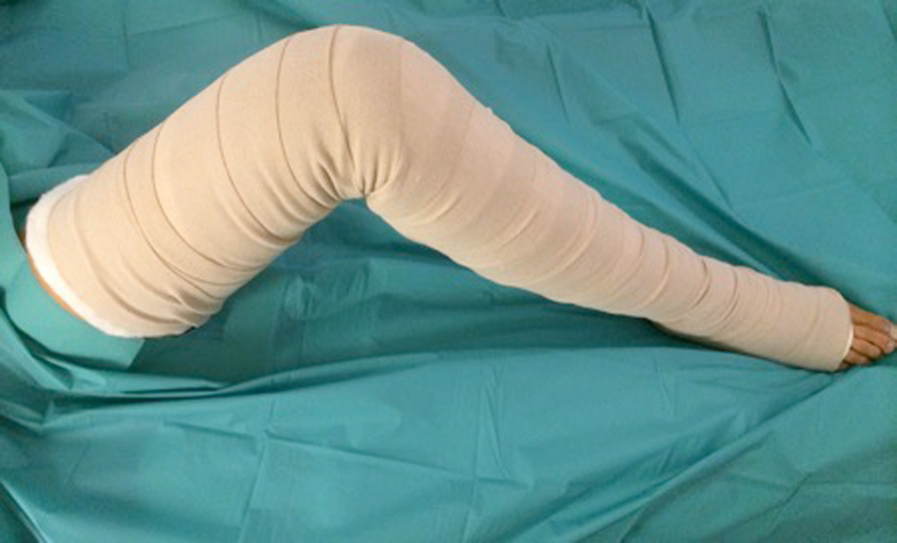
**Short-stretch, inelastic compression bandage.**

The compression bandage is applied over the hydrocolloid surgical wound dressing. A soft inner layer (Soffban, BSN Medical Ltd, Brierfield, UK) is applied from the toes to the groin on the affected leg with a 50% overlap of bandage. Following this the outer compressive layer bandage (Actico bandage, Activa Healthcare Ltd, UK) is applied firmly over the top, again with a 50% overlap of bandage. The bandage is pulled to full stretch before it is wrapped around the leg to ensure adequate compression in the application (Figure 2). A short-stretch, inelastic compressive bandage has been chosen because it is tolerable overnight due to its low resting pressure, yet produces high pressure with movement to greatly improve the efficacy of the calf muscle pump. The bandage is applied from the toes upwards. The application of bandage from thigh to groin requires removal of the tourniquet first and so the leg is kept elevated until the bandaging is complete. To ensure homogeneity in bandage application, the operating surgeons were shown a training video on correct application of the bandage and were given a tutorial on bandage application with real life bandage application and feedback. Correct bandage application was checked throughout the trial by the lead author and research nurse. The bandage is removed at 24 hours post-surgery leaving the hydrocolloid wound dressing *in-situ*. Otherwise the protocol follows that of the standard care group.

### Outcome measures

#### Study feasibility

Processes key to the future study will be analysed. Recruitment, retention and refusal rates will be calculated. The proportion of patients undergoing primary total knee arthroplasty who did not meet the eligibility criteria will also be calculated to ensure this is not too restrictive.

Resource allocation will be determined by calculating the time required for the recruitment and follow-up process. This will allow accurate resource allocation with regard to clinical research nurse and physiotherapy input.

Bandaging skill acquisition training video, ease of application and tolerance of the compression bandage will be determined from the operating surgeons and patients. Potential adverse risks associated with the use of the compression bandage such as pain, wound dehiscence and blistering will be recorded and rates calculated.

Qualitative feedback will be provided by the research team and clinical staff to determine any potential problems associated with the methodology with particular reference to the ease in obtaining the secondary outcome measures. The data will be analysed to calculate the completion rate of each outcome measure. A trial steering committee will meet before, half way through, and after the trial to discuss this feedback. The trial management group will complete day to day monitoring of the trial, consisting of the chief and principle investigator, the clinical research nurse and the research physiotherapists.

#### Feasibility of outcome measures

##### Swelling

Circumference or girth of the knee has been shown to have acceptable reliability for determination of gross changes in knee swelling in patients post-surgery [25]. Knee swelling is measured using the circumference of the knee at the mid-portion of the patella with the knee in full extension. Measurements are then taken 10 cm proximal to the superior pole of the patella (thigh) and 10 cm distal to the inferior pole of the patella (calf). The skin is marked at these points and the tape measure placed circumferentially around the limb, at and distal to the level of the mark). Measurements are in centimetres to one decimal place. Pre-operative values are taken on the day of surgery, every post-operative day after bandage removal until discharge and at 6-week follow-up.

##### Range of motion

Active range of motion at the knee is measured using a goniometer. The inter-tester reliability and validity of this method previously have been shown to be high [26]. The patient is positioned supine, with the hip and knee in the neutral position. The femur is stabilised to prevent rotation with a hand. The goniometer is placed on the lateral epicondyle of the femur, with the proximal arm on the lateral midline of the femur in line with the greater trochanter, and the distal arm on the lateral midline of the fibula in line with the lateral malleolus. Active flexion and extension values are recorded. Pre-operative values are taken on the day of surgery, every post-operative day after bandage removal until discharge and at 6-week follow-up.

##### Rest pain

At-rest pain scores from 0 to 10 using a visual analogue scale are assessed every day, prior to and after physiotherapy in hospital. The visual analogue scale is a simple and reliable method of recording pain and has been validated in lower limb arthroplasty [27]. Pre-operative values are taken on the day of surgery, and further values assessed on every post-operative day until discharge and at 6-week follow-up.

##### Patient reported outcome measures - knee pain and function

The Oxford knee score is a 12-point patient reported outcome questionnaire specifically designed and validated to assess function and pain after total knee arthroplasty [28]. Each question is scored out of 5 points. A total score is calculated and referenced to classify joint function as mild, moderate or severely affected by arthritis. This will be measured pre-operatively and at six months.

##### Patient reported outcome measures - health status

The Euroqol EQ-5D questionnaire is a descriptive system of health-related quality of life that can be applied to different health conditions [29]. It consists of five dimensions (mobility, self-care, usual activities, pain/discomfort, and anxiety/depression) each of which takes one of three responses for each dimension (no problems, some problems, or severe problems). This will be measured pre-operatively and at 6 months.

##### Length of stay

This is calculated from the patient’s admission and discharge dates and reflects midnights in hospital.

##### Readmissions and complications

Hospital episode statistic data is used to calculate readmission rates. Complications attributed to the compression bandage, wound complications and general medical complications within 30 days post-surgery will also be recorded.

### Confounding factors

Whilst confounding factors have been limited where possible, potential factors include age, sex, co-morbidities, pre-existing knee function, pre-existing chronic venous insufficiency, duration of operation, blood loss, time of surgery and compliance with rehabilitation. Pre-existing knee function and chronic venous insufficiency are however, in part, accounted for by taking pre-operative knee measurements.

### Statistical analysis

A secure Excel database will be used to record data (Microsoft Inc, Albuquerque, NM, USA). Statistical analysis will be performed using SPSS version 23.0 (SPSS Inc, Chicago, IL, USA).

A power calculation will be performed based on the mean and standard deviations of knee swelling values collected from this preliminary data to inform a larger, future trial. A treatment effect of a 3-cm reduction in swelling based on previous literature will be used, utilising a 1-cm error of measurement and a 2-cm clinical minimal relevance [19]. The risk of a type 1 error will be set at 0.05 and a type II error at 0.20. The retention rate from feasibility data will be incorporated into the final sample size number to allow for possible patient drop-out. The recruitment rate and resource allocation hours can then also be applied to the final sample size required to plan the future trial requirements.

For each outcome measure (knee swelling, range of motion, visual analogue score, EQ-5D score, Oxford knee score) the two groups will be compared at each applicable set time point (pre-operative, day 1, day 2, 6 weeks, and 6 months). The difference from pre-operative measurement will also be used to reduce the impact of confounding factors between groups. A 95% confidence interval of the group differences will be calculated for each variable. A Kolmogorov-Smirnov goodness of fit will be used to determine the distribution. If the distributions are equal, Student’s *t*-test for independent samples will be used. The Welch correction will be used if variances are unequal. If the distributions are unequal, the Mann-Whitney *U* test will be used. Significance will be denoted at *P* <0.05.

## Discussion

Enhanced recovery programmes in orthopaedic elective surgery have been shown to significantly improve patient length of stay and satisfaction [30]. The success of these programmes can be attributed to utilising evidence-based techniques and providing highly structured, co-ordinated care. Despite their success, pathways should constantly be scrutinised and updated in relation to current best practice. Post-operative knee swelling and pain is a potentially modifiable problem. It is associated with short-term morbidity and has an unknown effect on longer-term function [19].

Research regarding the use of compression bandages in knee arthroplasty has been limited by a paucity of robust, clinical data. Small studies have shown improvements in pain and range of motion, but further work is required [16,17]. There is large heterogeneity in protocols for total knee arthroplasty between surgeons and hospitals, and therefore, independent variables are often difficult to analyse. This trial aims to addresses this, by providing feasibility data to assess the appropriateness of the trial methodology for a future, larger trial, and by providing preliminary data regarding the efficacy of the compression bandage using both objective and subjective means.

We acknowledge limitations in the study. First, the pressure at which the bandage is applied cannot be standardised without the use of a pressure transducer, which would not be pragmatic for this trial. The bandage is designed however, to ‘lock out’ at the desired tension for application and, thus, will ensure similar application parameters between surgeons. Secondly, whilst visual analogue scales will be used to measure pain, it is not possible in this trial to standardise post-operative analgesia as this is dependent on the patients’ demands, allergies and co-morbidities. Finally, there is possible inter-operator variation in measurement of the objective outcomes such as range of motion and knee circumference. However, where possible, validated outcome measures have been utilised, and the methodology designed to reduce potential confounding factors.

## Trial status

The trial is currently recruiting patients. The first patients enrolled in the study are at the 6-month follow-up.

## Abbreviations

ABPI: ankle brachial pressure index
BMI: body mass index
TKA: total knee arthroplasty
